# Pasteurized blood samples for transfusion compatibility testing during the coronavirus disease 2019 outbreak

**DOI:** 10.1017/ice.2020.138

**Published:** 2020-04-16

**Authors:** Run Yao, Yamei Shen, Ying Tan, Pengcheng Zhou, Bijuan Li, Xuegong Fan, Ning Li

**Affiliations:** 1 Department of Blood Transfusion, Xiangya Hospital, Central South University, Changsha, 410007, Hunan, China; 2 Department of Infectious Diseases, Xiangya Hospital, Central South University, Changsha, China; 3 Key Laboratory of Viral Hepatitis, Hunan Province, Changsha, China

*To the Editor—*In December 2019, a novel coronavirus pneumonia (COVID-19) was reported in Wuhan, China. As of April 2, 2020, 82,774 confirmed cases had been reported in China and 874,995 confirmed cases had been reported in other countries. No vaccine or antiviral therapeutics are yet available to prevent or treat COVID-19.^1^ Preventing infection is the current priority for disease control.

The SARS-CoV-2 virus is transmitted from person to person through droplets or direct contact.^2^ However, non-respiratory samples are also potential sources of COVID-19 infection.^3^ Virus-laden aerosols generated from blood-sample centrifugation pose risks for laboratory staff and broader nosocomial transmission.^3,4^ Traditional precautionary measures for infectious-sample processing include tertiary protection and operating in the biological safety cabinet. Preventive resources have been limited during this multiregional outbreak, posing huge risks to laboratory staff. Therefore, effective methods to ensure the safety of laboratory staff in low-resource settings are needed.

Pasteurization at 56°C for 30 minutes has been recommended to inactivate coronavirus, which might decrease the infectivity of samples and aerosols. To reduce infections and ensure safe and effective transfusion, we investigated the effects of pasteurization on transfusion compatibility testing.

## Methods

Blood samples were collected from Xiangya Hospital, Central South University. Each sample was divided into 2 groups, an experimental group and a control group. Experimental samples were treated by pasteurization. The results of blood-group typing, irregular antibody screening, and cross-matching were compared between these 2 groups. Finally, samples of suspected SARS-CoV-2 were treated with pasteurization. Treated samples were used to test transfusion compatibility. Patients with suspected COVID-19 then received red blood cell (RBC) transfusion, and the effectiveness and safety of these transfusion were evaluated.

## Results

**Fig. 1.**
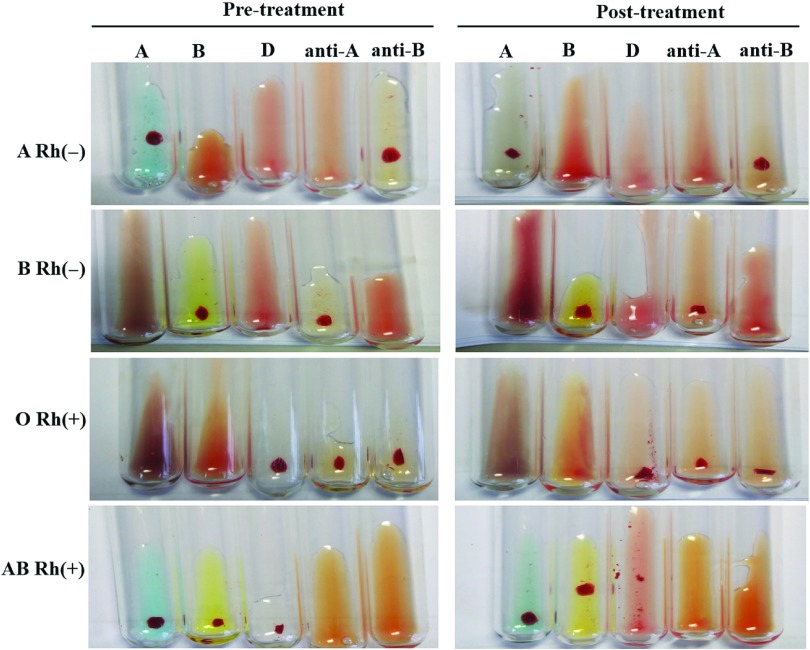
The results of blood group typing.

**Fig. 2.**
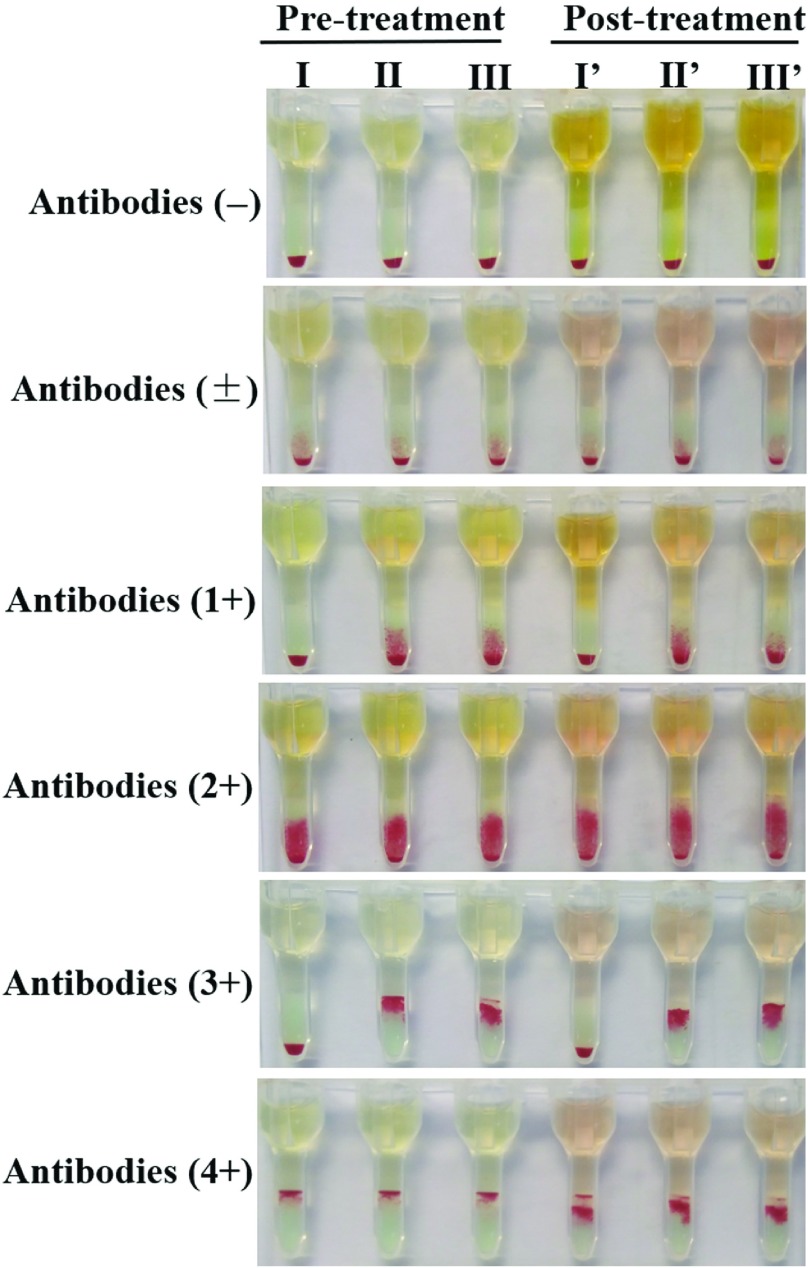
The results of irregular antibodies screening. I, II, III represent panel cells no. 1, no. 2, and no. 3, respectively (Shanghai Blood Biomedical, Shanghai, China).

The agglutination intensities of A, B antigens and anti-A, anti-B antibodies of the samples in the 2 groups were 4+. The forward and reverse types were consistent in the ABO blood group. In the Rh blood group, the agglutination intensity of D antigen was reduced from 4+ to between 2+ and 3+ after heat treatment (Fig. 1). Regarding the effect of heat treatment on irregular antibody screening, our results showed that the response pattern of panel cells remained unchanged after heat treatment when the agglutination intensity was negative(-), uncertain(±) or zero, and 1+, 2+, or 3+, respectively. However, the agglutination intensities of samples rating 4+ were reduced to 3+ after heat treatment (Fig. 2). Finally, no effect of heat treatment on the primary cross-matching was observed.

Our results indicated that heat treatment did not affect the results of transfusion compatibility testing. The RBC transfusion in patients were safe and effective based on elevated 24-hour hemoglobin results or improved symptoms, with no hemolytic reactions or other adverse transfusion reactions.^5^

## Discussion

We have demonstrated that pasteurization did not affect the results of transfusion compatibility testing and that blood transfusion based on this improved testing were safe and effective. Because the heat-inactivation method was simple, efficacious, and cost-effective, it could be employed for the protection of laboratory staff, especially in resource-poor regions during the COVID-19 pandemic.

Since virus activity testing was not available in our laboratory, we were unable to determine whether the virus can still be contagious after thermal inactivation. Reports indicated that SARS-CoV-2 was sensitive to heat and thermal inactivation could efficiently eliminate the coronavirus infectivity.^6^ Heat treatment causes RBCs to rupture and form RBC fragments, which may have affected the detection results. Especially in gel microcolumns, false-positive results are likely. Therefore, the classic test-tube method should be used to instead of blood-type cards to perform blood-group typing of the heat-treated samples. However, irregular antibody screening and cross-matching could be performed using the anti–human-globulin card method.

In conclusion, during the COVID-19 pandemic, pasteurization can be used to test transfusion compatibility, to protect laboratory staff from infected samples, and to ensure safe and effective transfusion. Moreover, pasteurization is convenient and quick and suitable for use in hospitals.
